# Dysregulated miRNA and mRNA Expression Affect Overlapping Pathways in a Huntington’s Disease Model

**DOI:** 10.3390/ijms241511942

**Published:** 2023-07-26

**Authors:** Nóra Zsindely, Gábor Nagy, Fruzsina Siági, Anita Farkas, László Bodai

**Affiliations:** 1 Department of Genetics, Faculty of Science and Informatics, University of Szeged, H-6726 Szeged, Hungary; zsindizsn@yahoo.com; 2 Department of Biochemistry and Molecular Biology, Faculty of Science and Informatics, University of Szeged, H-6726 Szeged, Hungary; 3 Doctoral School in Biology, Faculty of Science and Informatics, University of Szeged, H-6726 Szeged, Hungary

**Keywords:** Huntington’s disease, neurodegeneration, miRNA, transcriptomics, transcription, *Drosophila*, animal model

## Abstract

Huntington’s disease (HD) is a fatal neurodegenerative disorder caused by the expansion of a CAG trinucleotide repeat in the Huntingtin gene. Transcriptional dysregulation is one of the main cellular processes affected by mutant Huntingtin (mHtt). In this study, we investigate the alterations in miRNA and mRNA expression levels in a *Drosophila* model of HD by RNA sequencing and assess the functional effects of misregulated miRNAs in vivo. We found that in head samples of HD flies, the level of 32 miRNAs changed significantly; half of these were upregulated, while the other half were downregulated. After comparing miRNA and mRNA expression data, we discovered similarities in the impacted molecular pathways. Additionally, we observed that the putative targets of almost all dysregulated miRNAs were overrepresented among the upregulated mRNAs. We tested the effects of overexpression of five misregulated miRNAs in the HD model and found that while mir-10 and mir-219 enhanced, mir-137, mir-305, and mir-1010 ameliorated mHtt-induced phenotypes. Based on our results, we propose that while altered expression of mir-10, mir-137, and mir-1010 might be part of HD pathology, the upregulation of mir-305 might serve as a compensatory mechanism as a response to mHtt-induced transcriptional dysregulation.

## 1. Introduction

Huntington’s disease (HD, OMIM: #143100) is a fatal, late-onset neurodegenerative disorder caused by a dominant gain-of-function mutation in the *Huntingtin* (*HTT*, HGNC: 4851) gene. The disease-causing mutation is an expansion of a naturally occurring, polymorphic CAG trinucleotide repeat in the first exon of *HTT*. While CAG repeats shorter than 36 repeat units are not pathologic, repeats with 40 or more units cause disease with full penetrance [1]. The expanded CAG repeat is translated to an elongated polyglutamine repeat in the mutant Huntingtin (mHtt) protein that mediates aberrant protein interactions and aggregation, and induces a multifaceted pathomechanism [2,3], affecting diverse cellular processes, including the ubiquitin–proteasome system [4], signal transduction [5], mitochondrial impairment [6], and transcriptional regulation [7].

Besides dysregulation of protein-coding genes, altered expression of microRNA genes (miRNA) was also observed in HD [8,9]. miRNAs are endogenous regulatory short non-coding RNA molecules that are the end products of multistep processing of primary miRNA transcripts (pri-miRNA) transcribed, in most cases, by RNA polymerase II [10]. miRNAs are incorporated in the cytoplasmic RNA-induced silencing (RISC) complex that recognizes target mRNAs by complementary base pairing between the 3′-untranslated regions of mRNAs and the miRNA loaded into RISC. This ultimately leads to the repression of translation and mRNA degradation [11].

Dysregulated miRNA levels in HD contribute to the misregulation of gene expression networks [8,12,13,14] and can also influence mHtt levels as Huntingtin mRNA itself is targeted and regulated by miRNAs [15,16]. In this study, we aimed to identify miRNAs whose level is altered in a well-characterized *Drosophila melanogaster* model of HD [17] in which an N-terminal human mHtt fragment with 120 glutamines is expressed in the nervous system. Several characteristics of HD, e.g., neuronal degeneration, motor dysfunction, and lethality, are recapitulated in the *Drosophila* model and can be quantitatively characterized by determining the degeneration of photoreceptor neurons, measuring climbing speed, and determining viability and lifespan. Our results show that in response to mHtt, the level of 32 miRNAs changed significantly. Gene set enrichment analyses (GSEA) indicated a substantial overlap between the molecular pathways enriched among the putative targets of upregulated and downregulated miRNAs. Furthermore, by comparing miRNA-seq and mRNA-seq datasets, we found significant overlaps between dysregulated mRNAs and the putative targets of dysregulated miRNAs. To validate the pathology-related functional effects of dysregulated miRNAs, we performed genetic interaction screens and found that overexpression of *mir-137*, *mir-305*, and *mir-1010* ameliorate mHtt-induced pathology.

## 2. Results

### 2.1. Mutant Huntingtin Induces Specific Changes in miRNA Expression

To model HD in *Drosophila,* we expressed an N-terminal fragment of human Htt in the nervous system using the GAL4/UAS bipartite expression system [17]. The applied *UAS-HTTex1.Q120* (*Htt.Q120*) and *UAS-HTTex1.Q25* (*Htt.Q25*) transgenes carry the first exon of *HTT* with a pathological length (Q120) or normal length (Q25) polyglutamine domain, respectively. Both transgenes were integrated into the same genomic position to avoid positional effects [18]. To measure miRNA level changes in the HD model, we prepared small-RNA sequencing libraries from head samples of 5-day-old females expressing *Htt.Q120* (HD samples) or *Htt.Q25* (control samples) transgenes under the control of the pan-neuronal *P{GawB}elavC155* (*elav-GAL4*) driver and subjected them to Illumina sequencing. We analyzed eight biological replicates of HD and seven biological replicates of control small-RNA sequencing libraries with a median number of miRNA-specific reads of 1.04 million (Supplementary Table S1). The number of identified unique miRNAs in the head samples ranged between 176 and 211 (median: 199). Importantly, there was no significant difference in the diversity of the expressed miRNAs between HD and control samples (median values of 198.5 and 199, respectively).

**Figure 1:**
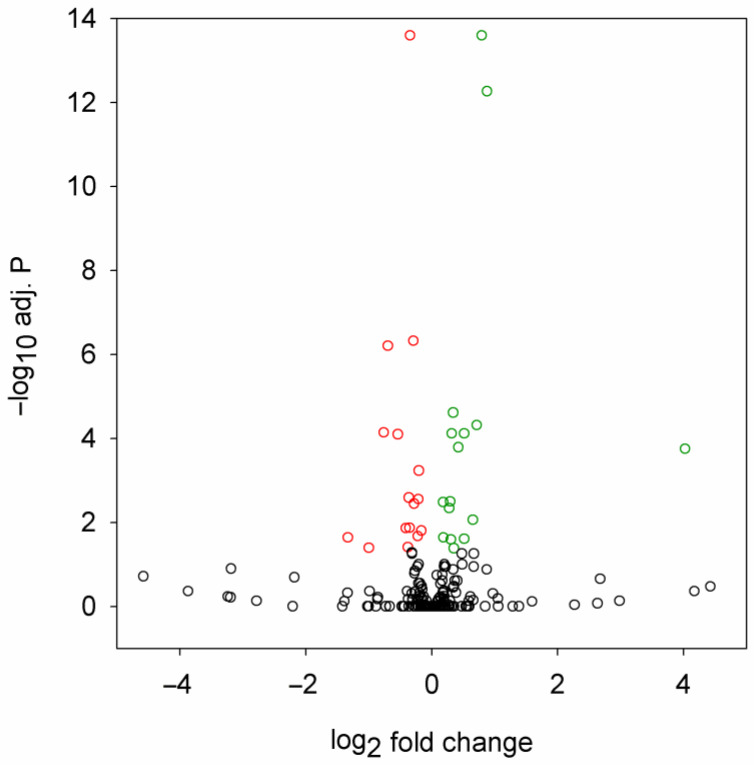
Volcano plot of differential expression analysis of miRNA sequencing data. Fold change is the ratio of miRNA expression level in *Htt.Q120* vs. *Htt.Q25* expressing flies. Significantly (adjusted *p* value < 0.05) downregulated or upregulated miRNAs are plotted with red or green, respectively.

By differential expression analysis, we identified 32 miRNAs that were significantly (adjusted *p* value < 0.05) misregulated in the HD flies (Figure 1, Table 1). In total, 16 of these were downregulated, while 16 were upregulated.

The observed change in the levels of most misregulated miRNAs was modest; the quantity of only three miRNAs changed by at least twofold (dme-miR-4986-3p and dme-miR-2279-5p were downregulated, dme-miR-961-5p was upregulated). The most significant hits with adjusted *p* < 10^−10^ were dme-miR-87-3p (downregulated), dme-miR-286-3p (upregulated), and dme-miR-4969-5p (upregulated).

To identify biological pathways that might be affected by changes in the levels of the identified miRNAs, we determined the predicted target genes of the affected miRNAs using TargetScanFly 7.2 [13] and then combined these predicted targets in target gene sets for further analysis (Supplementary File S1). To validate our model, first, we performed a GSEA to determine how the combined miRNA target gene set corresponds to differentially regulated gene sets described in the *Drosophila* models of human diseases. Seven out of the top ten disease terms with the most significant overlaps were related to neurodegenerative disorders (Supplementary Table S2), including the top three hits (tauopathy (overlap: 144/188, adjusted *p* = 1.52 × 10^−19^), HD (overlap: 111/136, adjusted *p* = 3.59 × 10^−19^), and Alzheimer’s disease (overlap: 77/87, adjusted *p* = 1.14 × 10^−17^), supporting the notion that the observed changes in miRNA levels are related to neurodegeneration.

**Figure 2:**
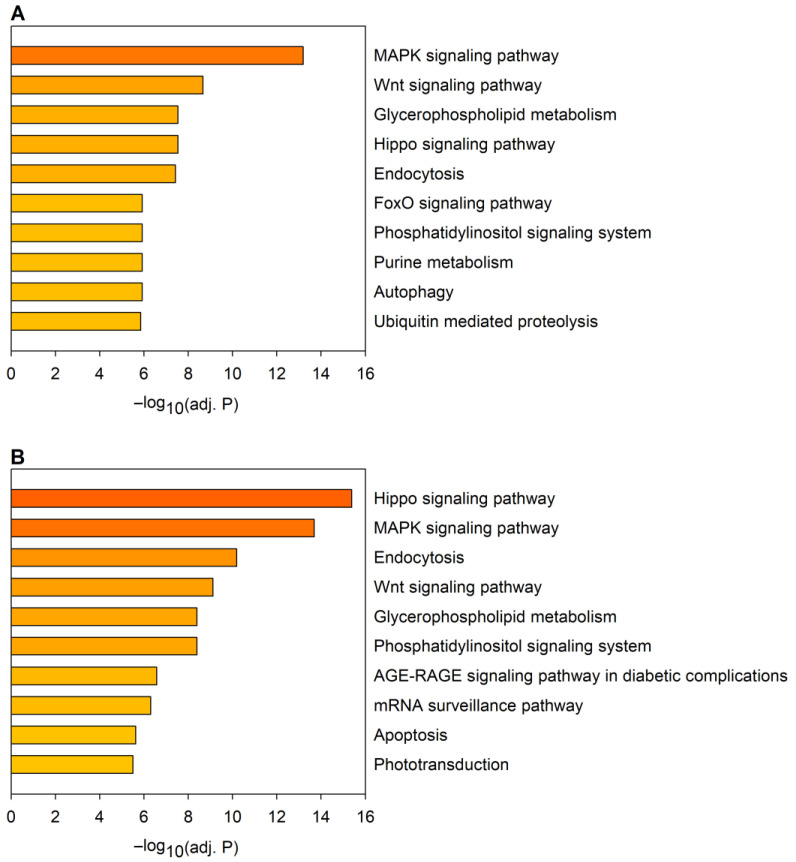
KEGG (Kyoto Encyclopedia of Genes and Genomes) pathway analysis of predicted target gene sets of miRNAs dysregulated in the Huntington’s disease (HD) model. The graph shows the top 10 most significantly enriched pathways in the combined target gene sets of (**A**) downregulated and (**B**) upregulated miRNAs. Statistical significance was calculated with Fisher’s exact test with Benjamini–Hochberg (BH) correction.

Next, we performed a KEGG (Kyoto Encyclopedia of Genes and Genomes) pathway analysis using the predicted target gene sets of downregulated and upregulated miRNAs (Figure 2). The analysis indicated that several of the significantly enriched pathways are affected in the target gene sets of both downregulated and upregulated miRNAs (Figure 2, Supplementary File S2). These KEGG pathways include the MAPK signaling pathway, the Wnt signaling pathway, the glycerophospholipid metabolism, the Hippo signaling pathway, endocytosis, the FoxO signaling pathway, the phosphatidylinositol signaling system, the AGE-RAGE signaling pathway in diabetic complications, and apoptosis, among others. As a substantial portion of the identified pathways is related to regulatory mechanisms, it is not surprising that the majority of the most significantly enriched gene ontology (GO) molecular function terms in the combined gene set of predicted miRNA targets are related to nucleic acid binding and transcription factor activity (8 out of the 10 most significant terms), and protein kinase activity (2 out of the 10 most significant terms) (Supplementary Table S3).

### 2.2. Correspondence between Dysregulated miRNA and mRNA Levels

**Figure 3:**
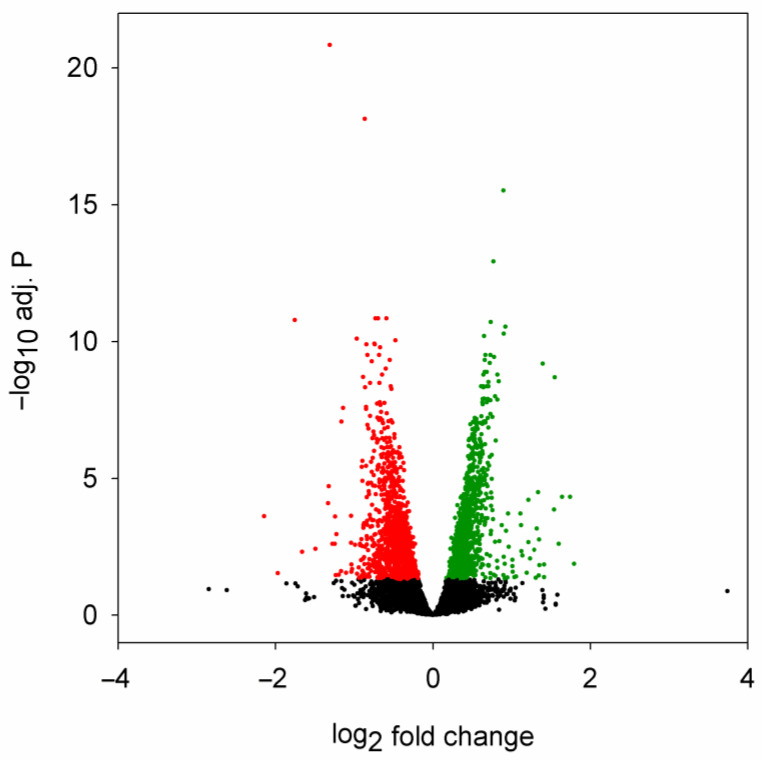
Volcano plot of differential expression analysis of mRNA sequencing data. Fold change is the ratio of mRNA expression level in *Htt.Q120* vs. *Htt.Q25* expressing flies. Significantly (adjusted *p* value < 0.05) downregulated or upregulated mRNAs are plotted with red or green, respectively.

To be able to characterize the potential connections between miRNA dysregulation and transcriptome-wide mRNA levels, we performed RNA sequencing on selected RNA samples used for miRNA sequencing after poly(A) selection (Supplementary Table S4). We found 2141 genes that were significantly dysregulated in the head samples of 5-day-old HD flies, 1056 of these genes were downregulated, while 1085 were upregulated (Figure 3, Supplementary File S3).

**Figure 4:**
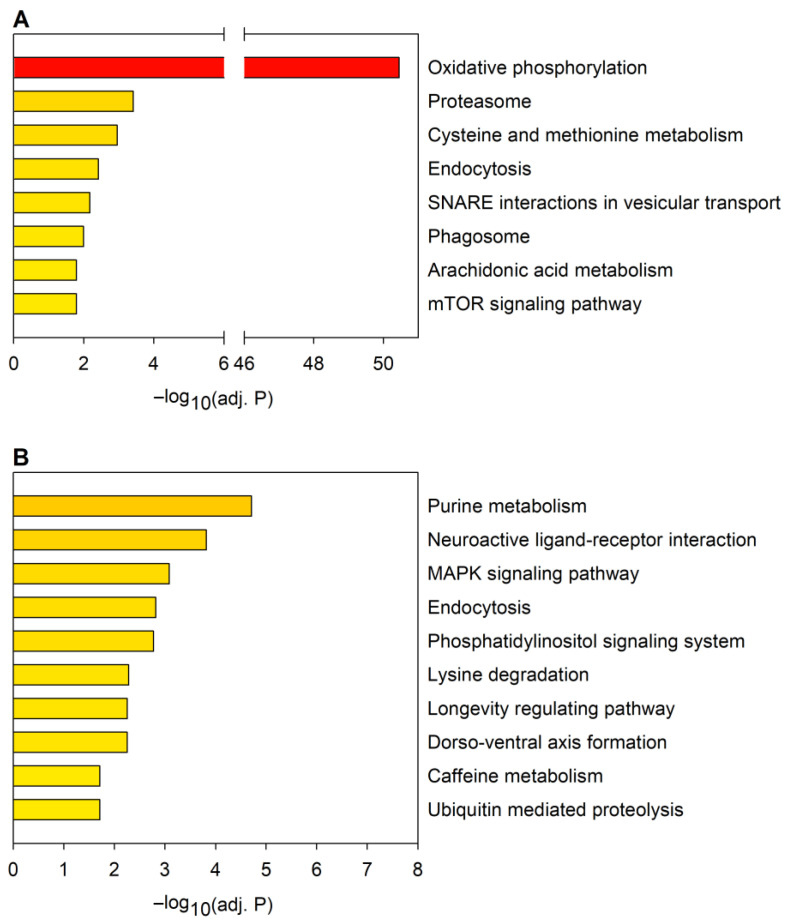
KEGG pathway analysis of mRNAs dysregulated in the HD model. The graph shows pathways significantly (*p* ≤ 0.05) enriched in the sets of (**A**) downregulated and (**B**) upregulated mRNAs. Statistical significance was calculated with Fisher’s exact test with Benjamini–Hochberg (BH) correction.

A GSEA of downregulated or upregulated mRNAs (Figure 4, Supplementary File S2) identified a smaller number of significantly enriched KEGG pathways (8 and 10 pathways in the down- and upregulated sets, respectively, at adjusted *p* ≤ 0.05) than similar analysis of the predicted mRNA target sets of dysregulated miRNAs (33 and 30 pathways in the down- and upregulated sets, respectively). In the case of the most enriched pathways, except for oxidative phosphorylation, the statistical significance of enrichments was less pronounced than what we have observed among the highest-ranking 2×10 enriched pathways in the target sets of dysregulated miRNAs.

KEGG pathways enriched in the downregulated mRNA set are ones related to metabolism, vesicular trafficking, proteasome, and mTOR signaling (Figure 4A). From these, the term phagosome was also enriched in the target set of downregulated miRNAs, while endocytosis was enriched in the targets of both the downregulated and upregulated miRNAs.

From the KEGG pathways enriched among upregulated mRNAs (Figure 4B), all except one (caffeine metabolism) were also significantly enriched among predicted targets of dysregulated miRNAs. The KEGG terms purine metabolism and ubiquitin-mediated proteolysis were enriched in the target set of downregulated miRNAs. Lysine degradation, longevity regulating pathway, dorso-ventral axis formation, neuroactive ligand–receptor interaction, phosphatidylinositol signaling system, MAPK signaling pathway, and endocytosis were enriched in the predicted target sets of both the downregulated and upregulated miRNAs. Thus, GSEA indicates that a substantial portion of the molecular pathways influenced by dysregulated mRNAs and miRNAs overlap.

**Figure 5:**
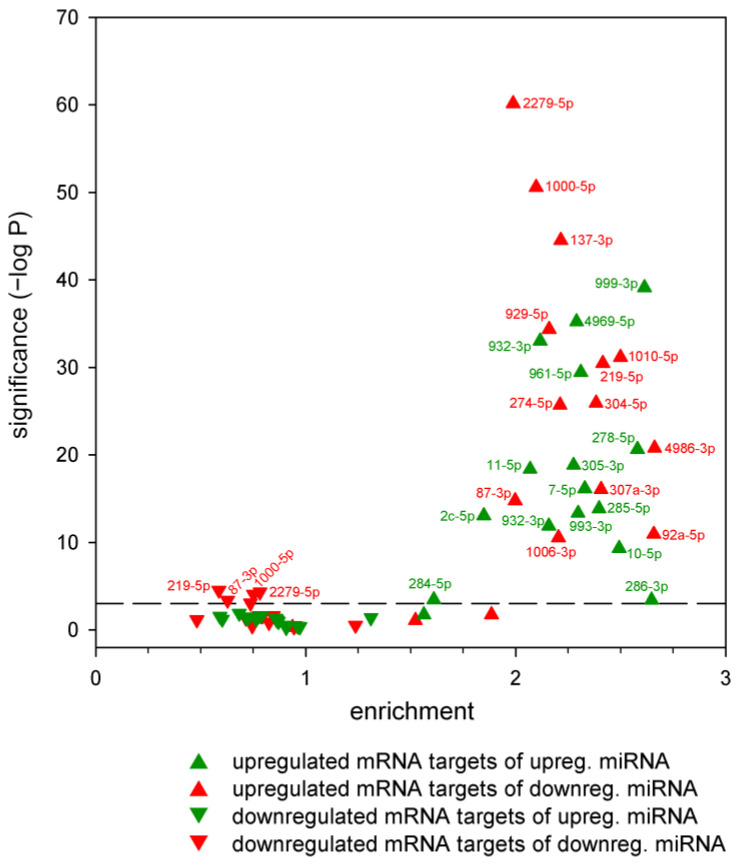
Enrichment analysis of putative targets of dysregulated miRNAs in the sets of up- and downregulated mRNAs in the HD model. Statistical significance of enrichment/depletion was calculated with hypergeometric test, the dashed reference line marks Bonferroni correction adjusted α = 0.05 level (7.81 × 10^−4^). Enrichment values < 1 correspond with depletion.

Next, we asked whether the predicted targets of each specific dysregulated miRNA were affected at the mRNA level. We applied a hypergeometric distribution test to establish whether putative targets of dysregulated miRNAs were over- or underrepresented among differentially expressed genes (Figure 5, Supplementary File S4). In this analysis, the following eight major outcomes were possible: the putative targets of down- or upregulated miRNAs could be enriched or depleted among down- or upregulated mRNAs. However, we found that only three outcome categories had significant entries. These are as follows: (1) the targets of a downregulated miRNA are underrepresented among downregulated genes (concerning miR-2279-5p, miR-219-5p, miR-87-3p, and miR-1000-5p); (2) the targets of a downregulated miRNA are overrepresented among upregulated genes (in the case of all downregulated miRNAs except miR-998-3p and miR-2b-3p); (3) the targets of an upregulated miRNA are overrepresented among upregulated genes (in the case of all upregulated miRNAs). Thus, among upregulated genes, the targets of nearly all miRNAs were overrepresented (irrespective of the change in miRNA level), while among downregulated genes, targets of only a few downregulated miRNAs were depleted. The change in target mRNA levels in categories (1) and (2) are consistent with a model in which dysregulated miRNA expression leads to reduced mRNA levels, considering that miRNAs exert their effects through both translational repression and mRNA decay [19]. In contrast, the change in mRNA levels in category (3) is not consistent with the suppressive role of miRNAs and indicates indirect and/or feedback effects.

### 2.3. The Effects of miRNA Overexpression in mHtt-Expressing Drosophila

We selected two miRNAs that were upregulated and three miRNAs that were downregulated in mHtt-expressing *Drosophila* to evaluate whether altering the levels of the identified miRNAs modifies the pathological phenotypes. For these analyses, we made crosses with flies carrying either the *Htt.Q120* or *Htt.Q25* transgenes producing the following four categories of progeny: females expressing both Htt and the specific miRNA (*elav-GAL4 UAS-Htt UAS-miR*), females expressing only Htt (*elav-GAL4 UAS-Htt*), and two non-expressing male categories without GAL4 driver (*UAS-Htt UAS-miR* and *UAS-Htt* non-expressing controls). Then, we analyzed the eclosion rate (the ratio of the number of females of a specific genotype and the number of the corresponding non-expressing male siblings), lifespan, vertical climbing speed, and neurodegeneration in the retina.

From the set of upregulated miRNAs, we selected *miR-10* and *miR-305* for testing. The putative targets of both miR-10-5p (enrichment (enr.) = 2.49×, *p* = 5.05 × 10^−10^) and miR-305-3p (enr. = 2.28×, *p* = 1.58 × 10^−19^) were significantly overrepresented among mRNAs upregulated in HD flies.

**Figure 6:**
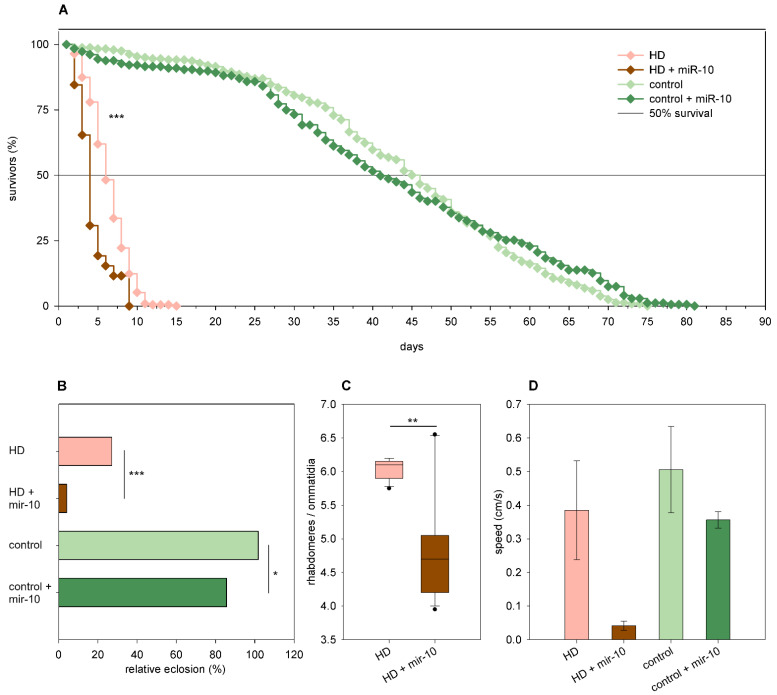
Overexpression of *mir-10* enhances mutant Huntingtin (mHtt) induced phenotypes. (**A**) Overexpression of *mir-10* decreases the longevity of *Htt.Q120* expressing (HD) flies. (**B**) *mir-10* overexpression leads to a significant decrease in the eclosion rate of both HD flies and *Htt.Q25* expressing controls. The graph shows the relative eclosion of HD and control flies in the presence or absence of a *mir-10* transgene as percent of non-expressing siblings. (**C**) Overexpression of *mir-10* decreases the number of visible rhabdomeres per ommatidia in the eyes of HD flies. The boxes show 25%, 50%, and 75% values, whiskers represent 10% and 90% values. (**D**) Overexpression of *mir-10* does not have a statistically significant effect on the climbing speed of either HD or control flies. Bars show average values, error bars indicate the standard error of mean (SEM). Significance levels (* *p* ≤ 0.05, ** *p* ≤ 0.01, *** *p* ≤ 0.001) are shown only between corresponding *mir-10* overexpressing and non-overexpressing categories.

Overexpression of *mir-10* had the following minor effects in *Htt.Q25* expressing flies: it resulted in a mild but statistically significant reduction of the eclosion rate (86% vs. 102% of wtHtt control, *p* = 0.023, Figure 6B), but it did not have a significant effect on median lifespan, neuronal survival, and climbing ability (Figure 6A,C,D). In *Htt.Q120* expressing females, however, *mir-10* overexpression had a negative effect on most analyzed phenotypes. It decreased the eclosion rate (4% vs. 27% of mHtt control, *p* = 9.9 × 10^−72^, Figure 6B) and longevity (*p* = 1.3 × 10^−7^, Figure 6A) of HD flies and reduced their median lifespan (3.44 vs. 5.87 days of mHtt control), although this effect was not significant. Furthermore, it enhanced the degeneration of photoreceptor neurons in 1-day-old flies (*p* = 0.01, Figure 6C) and led to a not-significant decrease in the speed of vertical climbing (Figure 6D).

**Figure 7:**
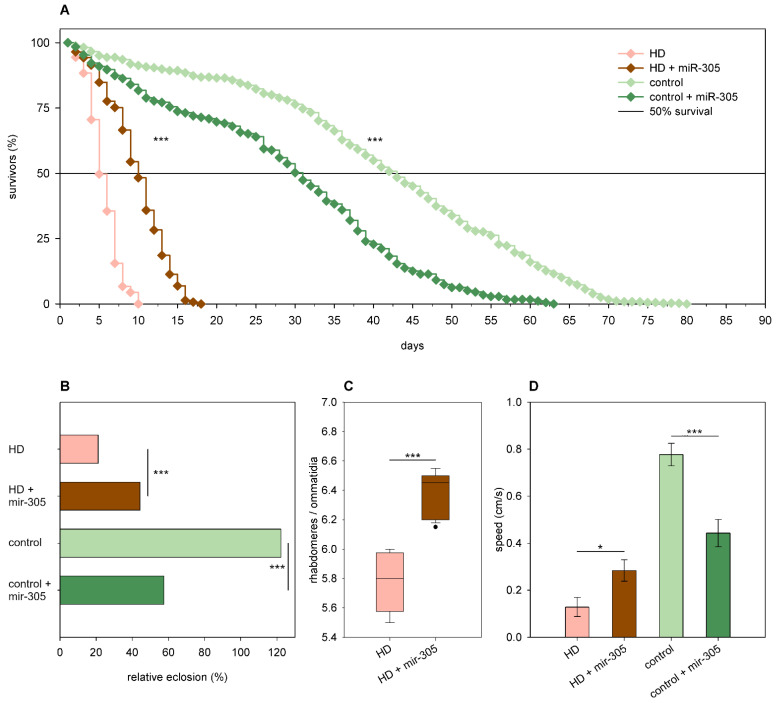
Overexpression of *mir-305* is detrimental to healthy flies but rescues mHtt-induced phenotypes. (**A**) Overexpression of *mir-305* decreases the longevity of *Htt.Q25* expressing (control) flies, while increases that of *Htt.Q120* expressing (HD) flies. (**B**) *mir-305* overexpression decreases the eclosion rate of *Htt.Q25* expressing flies, while it increases the eclosion rate of *Htt.Q120* flies. The graph shows the relative eclosion of flies expressing *Htt.Q120* (HD) or *Htt.Q25* (control) in the presence or absence of a *mir-305* transgene as percent of non-expressing siblings. (**C**) Overexpression of *mir-305* increases the number of visible rhabdomeres per ommatidia in the eyes of HD flies. The boxes show 25%, 50%, and 75% values, whiskers represent 10% and 90% values. (**D**) Overexpression of *mir-305* increases the climbing speed of HD flies, while decreases that of controls. Bars show average values, error bars indicate SEM. Significance levels (* *p* ≤ 0.05, *** *p* ≤ 0.001) are shown only between corresponding *mir-305* overexpressing and non-overexpressing categories.

In the case of *miR-305,* we found that, although its overexpression had negative effects in females expressing wtHtt, it rescued the phenotypes of HD flies. Thus, overexpression of *mir-305* decreased the eclosion rate (58% vs. 122% of wtHtt control, *p* = 4.7 × 10^−11^, Figure 7B), longevity (*p* < 10^−10^, Figure 7A), and median lifespan (30.09 days of 42.32 days of wtHtt control, *p* < 0.0003) of *Htt.Q25* expressing females, while it increased the eclosion rate (44% vs. 22% of mHtt control, *p* = 2.3 × 10^−7^, Figure 7B), longevity (*p* < 10^−10^, Figure 7A), and median lifespan (9.72 days of 4.99 days of mHtt control, *p* = 1.8 × 10^−12^) of *Htt.Q120* expressing females. Furthermore, we found that in 5-day-old *Htt.Q25* expressing flies, *mir-305* overexpression did not affect neuronal survival but decreased climbing speed (*p* = 0.0006, Figure 7D), while in 5-day-old *Htt.Q120* expressing flies, it ameliorated retinal neurodegeneration (*p* = 1.5 × 10^−6^, Figure 7C) and increased climbing speed (*p* = 0.011, Figure 7D).

From the set of downregulated miRNAs, we selected *miR-137*, *miR-219*, and *mir-1010* for testing. The putative targets of miR-137-3p (enr. = 2.22×, *p* = 3.19 × 10^−45^) and miR-1010-5p (enr. = 2.5×, *p* = 7.17 × 10^−32^) were significantly overrepresented among mRNAs upregulated in HD flies. The putative targets of miR-219-5p were significantly overrepresented among mRNAs upregulated in HD flies (enr. = 2.41×, *p* = 3.44 × 10^−31^) while significantly underrepresented among downregulated mRNAs (enr. = 0.59×, *p* = 2.94 × 10^−5^).

**Figure 8:**
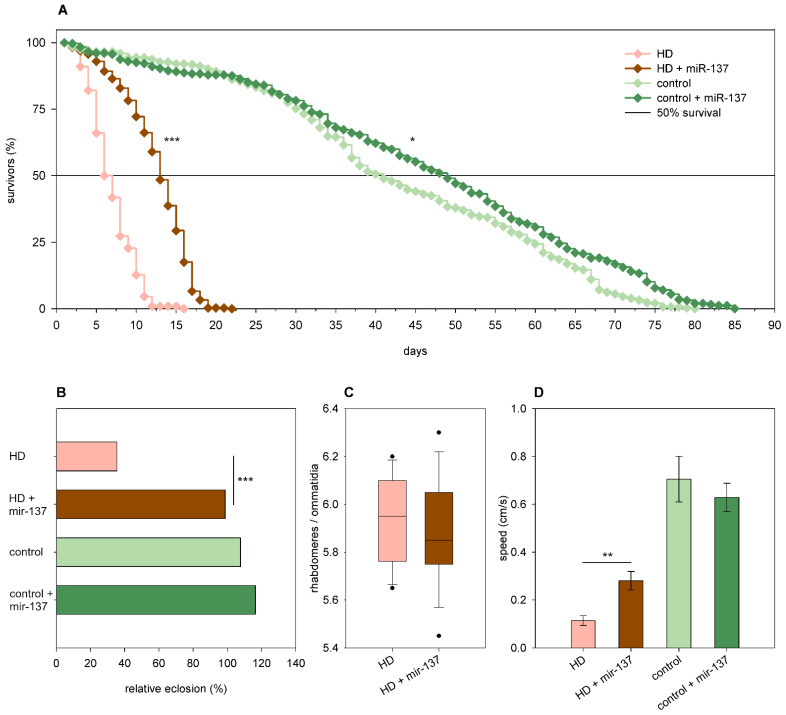
Overexpression of *mir-137* ameliorates mHtt-induced phenotypes. (**A**) Overexpression of *mir-137* increases the longevity of both *Htt.Q25* expressing (control) and *Htt.Q120* expressing (HD) flies. (**B**) *mir-137* overexpression leads to a significant increase in the eclosion rate of HD flies, while it does not affect the eclosion rate of controls. The graph shows the relative eclosion of flies expressing *Htt.Q120* or *Htt.Q25* in the presence or absence of a *mir-137* transgene as percent of non-expressing siblings. (**C**) Overexpression of *mir-137* does not affect the number of visible rhabdomeres per ommatidia in the eyes of HD flies. The boxes show 25%, 50%, and 75% values, whiskers represent 10% and 90% values. (**D**) Overexpression of *mir-137* increases the climbing speed of HD flies, while it does not have a similar effect on controls. Bars show average values, error bars indicate SEM. Significance levels (* *p* ≤ 0.05, ** *p* ≤ 0.01, *** *p* ≤ 0.001) are shown only between corresponding *mir-137* overexpressing and non-overexpressing categories.

Overexpression of *mir-137* in *Htt.Q25* females did not affect eclosion rate (Figure 8B), climbing speed (Figure 8D), and the survival of photoreceptor neurons; however, it led to a mild increase in longevity (*p* = 0.028, Figure 8A). In *Htt.Q120* expressing flies, it increased the eclosion rate (99% vs. 35% of mHtt control, *p* = 1.2 × 10^−36^, Figure 8B), longevity (*p* < 10^−10^, Figure 8A), and median lifespan (12.85 days vs. 6 days of mHtt control, *p* = 1.9 × 10^−12^). It did not affect the degeneration of photoreceptor neurons significantly in 5-day-old HD flies (Figure 8C) but increased climbing speed (*p* = 0.005, Figure 8D).

**Figure 9:**
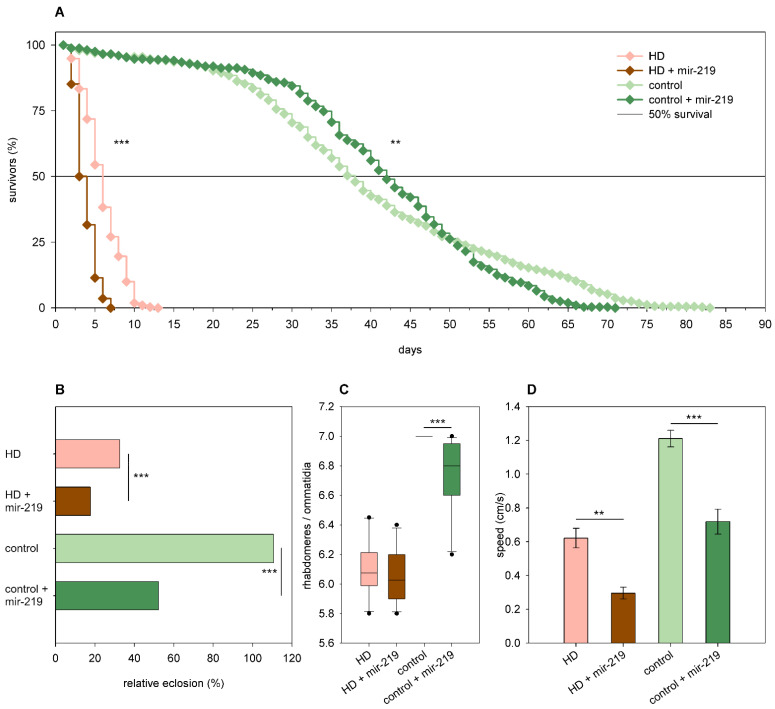
Overexpression of *mir-219*. (**A**) Overexpression of *mir-219* has a positive effect on the longevity of *Htt.Q25* expressing (control) flies, while a negative effect on the longevity of *Htt.Q120* expressing (HD) flies. (**B**) *mir-219* overexpression leads to decreased eclosion rate of both HD and control flies. The graph shows the relative eclosion of flies expressing *Htt.Q120* or *Htt.Q25* in the presence or absence of a *mir-219* transgene as percent of non-expressing siblings. (**C**) Overexpression of *mir-219* leads to a slight decrease in the number of rhabdomeres per ommatidia in control flies, but it does not affect neurodegeneration in the eyes of HD flies. The boxes show 25%, 50%, and 75% values, whiskers represent 10% and 90% values. (**D**) Overexpression of *mir-219* reduces the climbing speed of both HD flies and controls. Bars show average values, error bars indicate SEM. Significance levels (** *p* ≤ 0.01, *** *p* ≤ 0.001) are shown only between corresponding *mir-219* overexpressing and non-overexpressing categories.

Overexpression of *mir-219* led to a lower eclosion rate of both *Htt.Q25* (52% vs. 111% of wtHtt control, *p* = 4.32 × 10^−12^) and *Htt.Q120* (18% vs. 33% of mHtt control, *p* = 1.1 × 10^−4^) expressing flies (Figure 9B). *Mir-219* decreased the longevity (*p* < 10^−10^) and median lifespan of HD flies (3 days vs. 5.27 days in mHtt controls, *p* = 10^−12^) and also modified the shape of the survival curve of *Htt.Q25* expressing flies (*p* = 0.01) as follows: it increased the survival of younger adults but decreased that of older flies (Figure 9A). Furthermore, *mir-219* overexpression did not have a significant effect on neurodegeneration in 1-day-old HD flies, but it induced the loss of rhabdomeres in wtHtt-expressing flies (*p* = 5.4 × 10^−5^) (Figure 9C). Overexpression of *mir-219* resulted in reduced climbing speeds in 1-day-old flies expressing either wtHtt (*p* = 7.6 × 10^−4^) or mHtt (*p* = 2.6 × 10^−3^) (Figure 9D).

**Figure 10:**
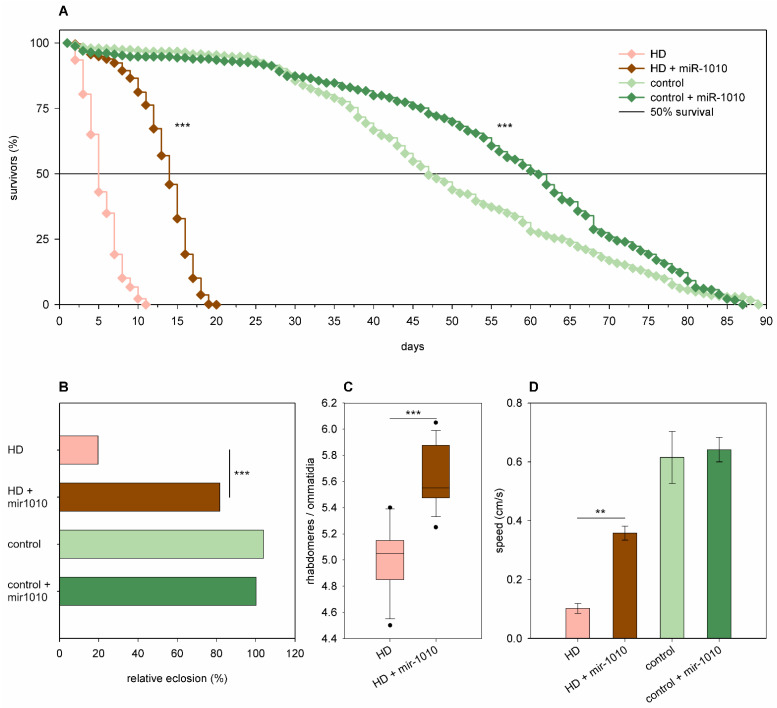
Overexpression of *mir-1010* ameliorates HD phenotypes. (**A**) Overexpression of *mir-1010* has a positive effect on the longevity of both *Htt.Q25* expressing (control), and *Htt.Q120* expressing (HD) flies. (**B**) *mir-1010* overexpression leads to increased eclosion rate of HD flies. The graph shows the relative eclosion of flies expressing *Htt.Q120* or *Htt.Q25* in the presence or absence of a *mir-1010* transgene as percent of non-expressing siblings. (**C**) Overexpression of *mir-1010* leads to an increase in the number of rhabdomeres per ommatidia in the eyes of HD flies. The boxes show 25%, 50%, and 75% values, whiskers represent 10% and 90% values. (**D**) Overexpression of *mir-1010* increases the climbing speed of HD flies. Bars show average values, error bars indicate SEM. Significance levels (** *p* ≤ 0.01, *** *p* ≤ 0.001) are shown only between corresponding *mir-1010* overexpressing and non-overexpressing categories.

Overexpression of *mir-1010* significantly increased the longevity (*p* = 6.8 × 10^−6^, Figure 10A) and median lifespan (60.86 days vs. 46.86 days of wtHtt control, *p* = 6 × 10^−9^) but did not affect the eclosion rate (Figure 10B), neuronal survival, and climbing speed (Figure 10D) in *Htt.Q25* expressing flies. In flies expressing *Htt.Q120*, however, it ameliorated every tested negative effect of mHtt at a significant level. *Mir-1010* overexpression increased the eclosion rate (82% vs. 20% in mHtt control, *p* = 1.53 × 10^−61^, Figure 10B), longevity (*p* < 10^−10^, Figure 10A), and the median lifespan (13.63 days vs. 4.69 days in mHtt control, *p* = 1.6 × 10^−12^) of HD flies, reduced the degeneration of photoreceptor neurons (*p* = 3.2 × 10^−6^, Figure 10C), and increased climbing speed (*p* = 0.0027, Figure 10D) in 5-day-old flies.

## 3. Discussion

### 3.1. Expression of Mutant Huntingtin Leads to miRNA Dysregulation

Huntington’s disease is one of several neurodegenerative disorders linked to, or caused by, protein misfolding and aggregation of aberrant proteins. Although the prevalence of HD is much lower than that of Alzheimer’s disease (AD) or Parkinson’s disease (PD), it became a prominent model of proteopathic neurodegenerative diseases because, due to its monogenic hereditary nature, it allows the generation of genetic animal disease models [20,21]. The extended polyglutamine domain in mutant Htt mediates aberrant protein interactions that culminate in complex pathogenesis with several affected cellular processes [3]. Transcriptional dysregulation is one of the major pathogenic processes in HD that was first characterized in detail by microarray analysis of striatal tissues of R6/2 and N171-82Q HD mouse models two decades ago [22]. Transcriptional alterations are present in HD mice before the onset of disease symptoms [23] and also in human asymptomatic HD mutation carrier individuals [24], suggesting that perturbed gene expression is not a mere consequence but one of the causes of pathogenesis.

In this study, we describe the results of a project aimed at identifying miRNAs that are misregulated in a *Drosophila* model of HD. As miRNAs function as post-transcriptional regulators of gene expression, their dysregulation introduces a second layer of altered gene regulatory mechanisms besides the transcriptional misregulation of mRNAs in HD and can pinpoint those molecular processes that are part of the response to mHtt-induced proteopathic stress. We found that the diversity of miRNAs expressed in *Drosophila* heads did not change in response to mHtt. Approximately 200 types of miRNAs were present at detectable levels, both in the HD and the control samples. However, the level of 32 miRNAs was altered in HD flies, half of them were upregulated, while the other half were downregulated. A GSEA of the putative targets of dysregulated miRNAs showed that the molecular processes in which the targets of downregulated or upregulated miRNAs are overrepresented have substantial overlap. Accordingly, 6 of the top 10 most affected KEGG pathways, including signaling pathways, were shared by downregulated and upregulated miRNAs.

As miRNAs mediate post-transcriptional regulation, we were interested to see if there are changes in the mRNA transcriptome that correspond to the dysregulated miRNAs. Therefore, we analyzed mRNA levels in some of the same samples by RNA-seq. The miRNAs affect mRNA function by repressing translation and/or inducing the degradation of target mRNAs. The latter of these effects directly influences the number of mRNAs and contributes to the quantitative changes in mRNA levels that can be detected by RNA-seq. Accordingly, the downregulation of a specific miRNA is expected to lead to increased cellular levels of its target mRNAs, while the upregulation of a specific miRNA is expected to result in decreased levels of its mRNA targets. However, it is also possible that the quantity of a specific miRNA is modulated as a secondary, compensatory response to the altered level of target mRNAs that are dysregulated at the level of transcription. In this case, increased levels of target mRNAs would lead to upregulation of miRNA expression, while decreased levels of target mRNAs would lead to downregulation of miRNA expression. When we analyzed the enrichment of the putative targets of the identified miRNAs among dysregulated mRNAs, we found examples for both scenarios. Targets of 13 out of the 16 downregulated miRNAs were overrepresented among upregulated miRNAs. Furthermore, targets of four of these miRNAs (dme-miR-2279-5p, dme-miR-219-5p, dme-miR-87-3p, and dme-miR-1000-5p) were also significantly underrepresented among downregulated genes. Thus, in the case of downregulated miRNAs, the changes in the expression levels of their targets did suit the model in which dysregulation of miRNAs is the primary effect, which is followed by corresponding changes in mRNA levels. In the case of upregulated miRNAs, however, targets of all but one miRNA were overrepresented among upregulated mRNAs, while none showed statistically significant enrichment or depletion among downregulated mRNAs. Thus, in the case of upregulated miRNAs, the changes in the expression levels of their targets did suit the alternative model in which dysregulation of mRNA transcription is the primary effect that is compensated by corresponding modulation of miRNA levels.

### 3.2. Overexpression of Specific miRNAs Alter mHtt-Induced Pathology

By performing genetic interaction tests in *Drosophila*, we determined the functional effects of selected miRNAs on mHtt-induced pathology. We tested the neuronal overexpression of three downregulated (*dme-mir-137*, *dme-mir-219*, and *dme-mir-1010*) and two upregulated (*dme-mir-10* and *dme-mir-305*) miRNA genes in the HD model. Based on the above-described logic, we assumed that reduced levels of dme-miR-137-3p, dme-miR-219-5p, and dme-miR-1010-5p are primary effects, while increased levels of dme-miR-10-5p and dme-miR-305-3p are consequences of a compensatory mechanism. However, we expected that miRNA overexpression might have positive effects in both the following cases: in the case of downregulated miRNAs by restoring normal miRNA levels, while in the case of upregulated miRNAs by further enhancing their compensatory effect. Accordingly, overexpression of two of the downregulated miRNAs, *dme-mir-137* and *dme-mir-1010,* ameliorated mHtt-induced phenotypes, indicating that the downregulation of these miRNAs is pathological. Overexpression of *dme-mir-219*, on the other hand, was detrimental both in the HD model and in control flies, suggesting that in this case, the observed phenotypical outcome is due to a toxic effect of *dme-mir-219* overexpression that is independent of HD pathology. Overexpression of *dme-miR-10* also exacerbated all analyzed phenotypes of HD flies, and it also had a mildly negative effect on the eclosion rate of control flies. This suggests that flies are sensitive to the level of dme-mir-10-5p, and increased expression of this miRNA might be a part of the molecular basis of pathology in the HD model. Finally, *dme-mir-305* overexpression alleviated all analyzed phenotypes of HD flies while it had significant detrimental effects on the viability, longevity, and motor activity of control flies. Similar effects on aging-related phenotypes were reported previously by Ueda et al., who showed that overexpression of *dme-mir-305* shortens lifespan, increases the loss of motor activity, and accelerates the accumulation of protein aggregates, while mir-305 depletion has the opposite effects [25]. The fact that *mir-305* overexpression ameliorates the phenotypes of HD flies even though it is detrimental to *wild-type* adults indicates that its positive effects are specific to the pathological processes induced by mHtt and might serve as a compensatory effect.

Two of the miRNAs whose overexpression led to enhanced HD pathology, *dme-miR-10* and *dme-miR-219*, and one of the miRNAs that ameliorated HD pathology, *dme-mir-137*, have strongly conserved human orthologs (Table 1) [18].

dme-miR-10, which was upregulated in our model and whose overexpression enhanced the HD pathology in flies, has several human homologs, including hsa-miR-10a, hsa-miR-10b, hsa-miR-99a, hsa-miR-100, and hsa-miR-146b. All of these orthologs were found to be dysregulated in the brain samples of HD patients. Hsa-mir-99b, hsa-mir-100, and hsa-mir-146a were found to be upregulated in the prefrontal cortical and striatal samples of HD patients, while hsa-miR-146b-5p was downregulated [9]. In another study analyzing miRNA levels in the prefrontal cortex, hsa-mir-10b-5p and hsa-mir-10b-3p were upregulated in HD patient samples [14]. Moreover, the expression levels of hsa-mir-10b-5p and hsa-mir-10b-3p were positively associated with CAG repeat length-adjusted striatal involvement and the Vonsattel grade of patients, while they were negatively associated with the CAG repeat length-adjusted age of disease onset [14]. Besides altered miRNA expression, altered miRNA editing that might alter the target profile of miRNAs was also observed in the prefrontal cortex of HD patients. In these samples, 129 significantly different miRNA editing events were identified, including increased modifications of hsa-mir-10a, hsa-mir-10b-5p, and hsa-mir-219a-2 [26].

Hsa-mir-219, the human ortholog of dme-mir219, enhanced HD pathology in flies in this study and is also implicated in several neurological disorders, including HD and AD. In post-mortem HD samples, hsa-miR-219b-3p was found to be downregulated in the prefrontal cortex [14]. In AD, the level of hsa-mir-219-5p was found to be downregulated in the brain tissues of patients undergoing radical resection [27], in the cerebrospinal fluid [28], and in the post-mortem cortical (Brodmann area 9) samples of patients suffering in AD or tangle-predominant dementia (TPD) [29]. mir-219 seems to modify AD pathology by regulating the quantity and phosphorylation of tau/MAPT protein. Mir-219-5p binds to the 3′-UTR of tau mRNA and directly represses tau expression both in human cells and in *Drosophila*. As a result, overexpression of *mir-219* in transgenic *Drosophila* expressing human tau protein reduced pathology, while its inhibition exacerbated it [29]. miR-219-5p was also found to affect the level of phosphorylated tau protein by directly regulating the levels of tau-tubulin kinase 1 (TTBK1) and glycogen synthase kinase 3β (GSK-3β). Accordingly, overexpression of miR-219-5p decreased, while its downregulation increased the levels of phosphorylated tau in cell culture [27].

Hsa-miR-137, the human ortholog of miR-137 that ameliorated HD pathology in flies, is enriched in brain tissues and has roles in neurogenesis and neuronal differentiation, and was implicated in several neurodegenerative disorders, including AD, PD, and HD [30]. Hsa-miR-137 was found to be downregulated in the frontal brain cortices of sporadic AD patients, and its expression showed a strong negative correlation with that of amyloid beta (Aβ) peptide [31]. In SH-SY5Y human neuroblastoma cells, miR-137 mimics were shown to inhibit Aβ-dependent hyperphosphorylation of the tau protein, while miR-137 inhibitors increased it [32]. Furthermore, in the astrocytes of transgenic mice expressing a mutant form of human amyloid precursor protein (APP), mir-137 overexpression resulted in the downregulation of endogenous Aβ, while its inhibition led to an increased Aβ level in a serine palmitoyltransferase long chain base subunit 1 (SPTLC1) dependent manner [31]. Hsa-miR-137 is increased in the blood plasma of PD patients [33], and inhibition of miR-137 in a mouse PD model resulted in reduced neuronal oxidative stress via upregulating the oxidation resistance 1 (OXR1) protein [34]. Dme-mir-137-3p was also found to be upregulated in an α-synuclein overexpression-based *Drosophila* PD model and regulated several members of the neuroactive-ligand receptor interaction pathway (dopamine receptor (D2R), γ-aminobutyric acid receptor (GABA-B-R3), and N-methyl-D-aspartate receptor (Nmdar2) dysregulated in PD flies [35]. In relation to HD, hsa-miR-137 was found to be downregulated in the striatum of HD patients [9], and it directly affects Huntingtin level by binding to the 3′-UTR of HTT mRNA and downregulating its level [36].

In conclusion, we found that similarly to the human HD condition, dysregulation of miRNAs can be observed in the fly model. We observed a substantial overlap between the putative targets of dysregulated miRNAs and dysregulated mRNAs in the model, suggesting that specific cellular processes are (mis)regulated at several levels upon proteopathic stress. Furthermore, our data suggest that miRNAs, on the one hand, might be primary targets of transcriptional dysregulation, while on the other hand, they can be a part of a feedback mechanism induced by the dysregulated mRNA expression. Importantly, using genetic interaction tests, we showed that altering the levels of specific dysregulated miRNAs influences HD pathology in flies. Based on these observations, fly models of HD could be useful tools to investigate miRNA-regulated processes upon proteopathic stress.

## 4. Materials and Methods

### 4.1. Drosophila Stocks and Crosses

*Drosophila* stocks were maintained and crosses were performed at 25 °C on standard *Drosophila* medium. The *w; UAS-HTTex1.Q25* and *w; UAS-HTTex1.Q120* [17] transgenic strains were donated by J. Lawrence Marsh (University of California Irvine, USA). The w^*^; *M{w^+mC^ = UAS-mir-10.Sb}ZH-86Fb/TM3*, *Sb^1^ Ser^1^*, w^*^; *M{w^+mC^ = UAS-mir-137.S}ZH-86Fb*, w^*^; *M{w^+mC^ = UAS-mir-219.Sb}ZH-86Fb, w^1118^*; *P{y^+t7.7^ w^+mC^ = UAS-LUC-mir-305.T}attP2/TM3*, *Sb^1^*, and w^*^; *P{y^+t7.7^ w^+mC^ = UAS-mir-1010.S}attP2*/*TM3, Sb^1^ Ser^1^* UAS lines and the *w P{GawB}elavC155* (henceforth *elav-GAL4*) driver line was from the Bloomington Drosophila Stock Center (Bloomington, IN, USA).

To generate flies expressing an HTT exon1 construct with normal length (Q25) or elongated (Q120) polyglutamine domain *elav-GAL4* females were mated with *w; UAS-HTTex1.Q25* or *w; UAS-HTTex1.Q120* males, respectively. For further analysis, freshly eclosed male and female progeny were collected and kept in separate vials while passing into fresh vials every second day.

To analyze the effects of miRNA overexpression on HD phenotypes, males carrying the miRNA overexpressing UAS constructs were first mated with *elav-GAL4*; *Sb*/*TM6* females then *elav-GAL4*; *UAS-miRNA*/*Sb* males were crossed to *w; UAS-HTTex1.Q120* (HD) or *w; UAS-HTTex1.Q25* (wtHtt control) virgins. Females (*elav-GAL4*/*w*; *UAS-HTTex1*/*+*; *UAS-miRNA*/+ and *elav-GAL4*/*w*; *UAS-HTTex1*/*+*; *Sb*/+) derived from these crosses carry the *elav-GAL4* driver and express UAS transgenes while males (*w*; *UAS-HTTex1*/*+*; *UAS-miRNA*/+ and *w*; *UAS-HTTex1*/*+*; *Sb*/+) are non-expressing controls. To analyze the effects of the tested miRNAs on the viability of *Htt*-expressing flies the number of progeny belonging to these four genotype categories were counted for 5 days after the beginning of eclosion and the relative eclosion rates of transgene-expressing female categories were expressed in percent of non-expressing male siblings. The numbers of flies analyzed in these crosses were the following: *mir-10 Q120*: 5108, *mir-10 Q25*: 2773, *mir-137 Q120*: 2852, *mir-137 Q25*: 1798, *mir-219 Q120*: 1077, *mir-219 Q25*: 1508, *mir-305 Q120*: 1138, *mir-305 Q25*: 1287, *mir-1010 Q120*: 3208, and *mir-1010 Q25*: 1997.

### 4.2. RNA Preparation

Five-day-old flies were frozen in liquid N_2_ and kept at −80 °C until sample preparation. Heads of frozen flies were removed by vortexing then total RNA samples were prepared from the collected heads using NucleoSpin miRNA kit (Macherey-Nagel, Düren, Germany) following the manufacturer’s protocol. RNA concentration and integrity were determined with a 2100 Bioanalyzer (Agilent, Santa Clara, CA, USA) capillary gel electrophoresis instrument using RNA 6000 Nano Kit (Agilent). Samples of RIN ≥ 7 were used for further analysis.

### 4.3. Small-RNA Sequencing and RNA Sequencing

Small-RNA sequencing libraries were prepared from 1000 ng total RNA samples using NEBNext Multiplex Small RNA Library Prep Kit for Illumina (New England Biolabs (NEB), Ipswich, MA, USA) following the manufacturer’s protocol with modifications. Modifications included addition of a *Drosophila*-specific custom blocking oligo mix (ACAACCCTCAACCATATGTAGTCCAAGCA, ATGAGCCGAGTGATCCACCGCTTAGAGTT, and GGAATTGGAACCGTATTCCCTTTCGTTCAAAATTAT at 80 nM, 40 nM, and 40 nM working concentrations, respectively) along with the SR RT primer for Illumina to the 3′ SR adaptor-ligated RNAs, 2× dilution of the 5′ SR adapter with nuclease-free H_2_O, and PCR amplification for 15 cycles. Size selection of library fragments after PCR amplification was performed using AMPure XP Beads (Beckman Coulter, Brea, CA, USA) as described in point 6C of the NEBNext protocol. Indexed small-RNA sequencing libraries were validated and quantitated using DNA 1000 kit (Agilent) in a 2100 Bioanalyzer (Agilent) instrument. Libraries were pooled, denatured with 0.2 M NaOH (MilliporeSigma, Burlington, MA, USA), and loaded in MiSeq Reagent Kit V3-150 (Illumina, San Diego, CA, USA) at a concentration of 16 pM. Sequencing was performed with an MiSeq (Illumina) instrument using the FASTQ-only workflow and generating 50 bp single-end sequence reads. Sequencing was performed on seven Htt.Q25 and eight Htt.Q120 biological replicates, the average number of raw sequence reads (±SD) was 2.95 ± 0.61 million and 3.04 ± 0.49 million, respectively.

For mRNA sequencing, polyA-RNAs were selected from 1000 ng total RNA samples using NEBNext Poly(A) mRNA magnetic isolation module (NEB) then strand-specific, indexed RNA-seq libraries were prepared using NEBNext Ultra II Directional RNA Library Prep Kit for Illumina (NEB) with NEBNext Multiplex Oligos for Illumina (NEB) following the recommendations of the manufacturer. Indexed RNA sequencing libraries were validated and quantitated using DNA 1000 (Agilent) kit in a 2100 Bioanalyzer (Agilent) instrument. The libraries were pooled, then denatured with 0.2 M NaOH and loaded in MiSeq Reagent Kit V3-150 (Illumina) at a concentration of 16 pM. Sequencing was performed with a MiSeq (Illumina) instrument using the FASTQ-only workflow generating 2x75 bp paired-end sequence reads. Sequencing was performed on four biological replicates per genotype, the average number of reads (±SD) was 6.37 ± 0.76 million and 6.34 ± 0.37 million in the case of Htt.Q25 and Htt.Q120 samples, respectively. Base-calling, BCL to FASTQ conversion, and demultiplexing were performed by default BaseSpace Sequence Hub (https://basespace.illumina.com, accessed on 4 February 2021) algorithms.

### 4.4. Secondary Sequence Analysis

To analyze miRNA-seq data, FASTQ files were quality checked with FastQC v0.11.9 (Babraham Institute, Cambridge, UK) [37] then adapter sequences matching the AGATCGGAAGAGCACACGTCTGAACTCCAGTCAC string were trimmed with Cutadapt v1.17 (National Bioinformatics Infrastructure Sweden, Uppsala, Sweden) [38]. Sequence reads of length ≥ 15 bases were aligned to bowtie-indexed reference sequences with miRge v2.0 (Johns Hopkins University, Baltimore, MD, USA) [39] and read counts were determined. Read count data were imported to R v4.0.2 (R Core Team) and differential expression analysis was performed with DESeq2 v1.26.0 (University of North Carolina, Chapel Hill, NC, USA) [40] applying likelihood ratio test for hypothesis testing (using parameters: test = “LRT”, reduced = ~1). miRNAs for which the counts-per-million (CPM) value was not >1 in at least 3 samples were filtered out. To adjust *p* values for multiple comparison testing Benjamini–Hochberg procedure (B-H) was applied. Putative RNA targets of the miRNAs showing altered levels were identified using TargetScanFly v7.2 (Whitehead Institute, Cambridge, MA, USA) [41] and after merging gene list enrichment analysis was performed using FlyEnrichr (Icahn School of Medicine at Mount Sinai, New York City, NY, USA) [42].

To analyze RNA-seq data, FASTQ files were quality checked with FastQC v0.11.9 then quality trimmed with Trim Galore v0.6.5 (Babraham Institute, Cambridge, UK). Sequence reads of length ≥ 36 bases were aligned to the *Drosophila melanogaster* r.6.37 reference genome with HISAT2 v2.2.0 (University of Texas, Dallas, TX, USA) [43] and gene-specific read counts were determined using the summarizeOverlaps function of the GenomicAlignments Bioconductor v3.11 (Fred Hutchinson Cancer Center, Seattle, WA, USA) package. Differential expression analysis was performed with DESeq2 v1.26.0, *p* values were adjusted with Benjamini–Hochberg procedure (B-H) for multiple comparison testing. GSEA was performed using FlyEnrichr [42].

### 4.5. Lifespan Analysis

Freshly eclosed *elav-GAL4*/*w*; *UAS-HTTex1.Q120*/*+*; *UAS-miRNA*/*+*, *elav-GAL4*/*w*; *UAS-HTTex1.Q120*/*+*; *Sb*/*+*, *elav-GAL4*/*w*; *UAS-HTTex1.Q25*/*+*; *UAS-miRNA*/*+*, and *elav-GAL4*/*w*; *UAS-HTTex1.Q25*/*+*; *Sb*/*+* females were transferred to fresh vials (maximum 30 flies per vial) and kept at 25 °C. Flies were passed to fresh vials on every second or third day, the number of deceased flies was recorded daily. The average number of flies analyzed was 333 per genotype (range: 39–706).

### 4.6. Analysis of Neurodegeneration

Neurodegeneration was monitored in the eyes of *elav-GAL4*/*w*; *UAS-HTTex1.Q120*/*+*; *UAS-miRNA*/*+*, *elav-GAL4*/*w*; *UAS-HTTex1.Q120*/*+*; *Sb*/*+*, *elav-GAL4*/*w*; *UAS-HTTex1.Q25*/*+*; *UAS-miRNA*/*+*, and *elav-GAL4*/*w*; *UAS-HTTex1.Q25*/*+*; *Sb*/*+* females by counting the number of rhabdomeres, light-gathering structures of photoreceptor neurons, in ommatidia of the compound eyes via the pseudopupil assay [44]. In wild-type flies there are always seven visible rhabdomeres in each ommatidium, smaller numbers indicate the degeneration of photoreceptor neurons. The heads of anesthetized flies were removed using a razor blade and they were fixed in drops of clear nail polish on microscope slides. The mounted heads were covered with immersion oil (Merck, Rahway, NJ, USA) then the rhabdomeres in the eyes were visualized using a Nikon Eclipse 80i (Nikon, Tokyo, Japan) compound microscope with 50× oil immersion objective immediately after mounting. We counted the number of visible rhabdomeres of at least 20 ommatidia in the eyes of at least 8 flies per genotype and recorded these numbers in data tables. For descriptive statistics, first, we calculated the average number of rhabdomeres per ommatidia for each fly of a given genotype, then calculated the mean of these averages.

### 4.7. Analysis of Motor Performance

Motor performance of *elav-GAL4*/*w*; *UAS-HTTex1.Q120*/*+*; *UAS-miRNA*/*+*, *elav-GAL4*/*w*; *UAS-HTTex1.Q120*/*+*; *Sb*/*+*, *elav-GAL4*/*w*; *UAS-HTTex1.Q25*/*+*; *UAS-miRNA*/*+*, and *elav-GAL4/w*; *UAS-HTTex1.Q25*/*+*; *Sb*/*+* females was monitored by climbing assay. The 1-day-old or 5-day-old flies were transferred to empty glass vials. The vials were gently tapped to a soft horizontal surface to knock all flies to the bottom then their movement was recorded on video for 10 s with recording speed of 30 frames per second. This procedure was repeated six times for each vial. Video recordings were analyzed and the average speed of flies in a measurement were determined with the FreeClimber v0.3.2 (Brown University, Providence, RI, USA) software [45]. Median values of climbing speed for each vial were calculated based on the average speeds of the six measurements. On average 155 flies (range: 12–430) in 3–16 vials were analyzed per genotype.

### 4.8. Statistical Analysis

Eclosion rates were calculated by dividing the number of females in an experimental category by the number of corresponding non-expressing control male siblings. Χ^2^-test was used to evaluate the relation of the *Htt* expression and the miRNA expression in the eclosion data. Survival data were analyzed using the Oasis 2 (Pohang University of Science and Technology, Pohang, South Korea) application [46]. The statistical differences of survival curves were evaluated with Peto-Peto-Prentice Test. The median survival times were calculated by linear interpolation of mortality curves and statistical significance was evaluated using Fisher’s exact test. For the analysis of pseudopupil data, the average number of rhabdomeres per ommatidia were calculated for at least 8 eyes per genotype and pair-wise comparisons were tested using Wilcoxon rank-sum test. The statistical significance of climbing assays was evaluated using Kruskal–Wallis Test with Bonferroni correction followed by Mann–Whitney U post-hoc tests.

## Figures and Tables

**Table 1:** miRNAs significantly misregulated in flies expressing mutant Huntingtin.

miRNA	Base Mean (CPM) ^1^	log_2_(FC) ^2^	Adjusted *p* Value ^3^	Human Orthologs ^4^
dme-miR-4986-3p	6.02	−1.33	2.29 × 10^−2^	
dme-miR-2279-5p	5.21	−1	4.03 × 10^−2^	
dme-miR-219-5p	23.94	−0.76	7.21 × 10^−5^	hsa-miR-219-5p ^$#^
dme-miR-1010-5p	25.86	−0.7	6.29 × 10^−7^	hsa-miR-412 ^#^
dme-miR-92a-5p	39.12	−0.54	8.01 × 10^−5^	hsa-miR-25 ^$#^, hsa-hsa-miR-92a ^$#^, hsa-miR92b ^$#^, hsa-miR-32 ^#^, hsa-miR-363 ^#^, hsa-miR-367 ^#^, hsa-miR-885-5p ^#^
dme-miR-307a-3p	213.68	−0.42	1.37 × 10^−2^	
dme-miR-304-5p	27.69	−0.38	3.85 × 10^−2^	hsa-miR-216a ^$#^
dme-miR-929-5p	117.03	−0.36	2.60 × 10^−3^	
dme-miR-274-5p	1271.69	−0.35	1.35 × 10^−2^	hsa-miR-758 ^#^
dme-miR-87-3p	199.18	−0.35	2.54 × 10^−14^	
dme-miR-1006-3p	198.74	−0.29	4.75 × 10^−7^	
dme-miR-137-3p	934.73	−0.28	3.60 × 10^−3^	hsa-miR-137 ^$#^
dme-miR-998-3p	158.26	−0.22	2.12 × 10^−2^	hsa-miR-21* ^#^, hsa-miR-29a ^#^, hsa-miR-29b ^#^, hsa-miR-29c ^#^, hsa-miR-593* ^#^
dme-miR-2b-3p	2802.63	−0.21	2.79 × 10^−3^	hsa-miR-499-3p ^#^
dme-miR-1000-5p	936.13	−0.21	5.86 × 10^−4^	
dme-miR-13b-3p	2027.42	−0.16	1.57 × 10^−2^	hsa-miR-499-3p ^#^
dme-miR-11-5p	256.11	0.18	3.29 × 10^−3^	hsa-miR-27b ^$#^, hsa-miR-27a ^#^, hsa-miR-128 ^#^, hsa-miR-499-3p ^#^, hsa-miR-768-3p ^#^
dme-miR-999-3p	79,593.87	0.19	2.28 × 10^−2^	
dme-miR-927-5p	5481.4	0.28	4.59 × 10^−3^	
dme-miR-932-3p	89.88	0.29	3.17 × 10^−3^	
dme-miR-993-3p	306.58	0.3	2.56 × 10^−2^	hsa-miR-100* ^$^, hsa-mir-99a* ^#^, hsa-miR-99b* ^#^, hsa-miR-556-5p ^#^
dme-miR-284-5p	1428.7	0.31	7.61 × 10^−5^	
dme-miR-285-5p	309.28	0.34	2.44 × 10^−5^	hsa-miR-29a ^$#^, hsa-miR-29b ^$#^, hsa-miR-29c ^$#^, hsa-miR-21* ^#^, hsa-miR-593* ^#^
dme-miR-7-5p	52,010.66	0.35	4.16 × 10^−2^	hsa-miR-7 ^$#^, hsa-miR-9* ^#^, hsa-miR-548-3p ^#^, hsa-miR-146a ^#^, hsa-miR-146b-5p ^#^
dme-miR-2c-5p	214.24	0.42	1.62 × 10^−4^	hsa-miR-499-3p ^#^
dme-miR-10-5p	1393.19	0.52	2.47 × 10^−2^	hsa-miR-10a ^$#^, hsa-miR-10b ^$#^, hsa-miR-99a ^$^, hsa-miR-100 ^$^, hsa-miR-146b-3p ^#^
dme-miR-278-5p	618.26	0.52	7.61 × 10^−5^	
dme-miR-6-3p	70.38	0.65	8.73 × 10^−3^	hsa-miR-27a ^#^, hsa-miR-27b ^#^, hsa-miR-128 ^#^, hsa-miR-499-3p ^#^, hsa-miR-768-3p ^#^
dme-miR-305-3p	487.46	0.71	4.85 × 10^−5^	
dme-miR-286-3p	16.1	0.79	2.54 × 10^−14^	hsa-miR-134 ^#^
dme-miR-4969-5p	31.1	0.88	5.42 × 10^−13^	
dme-miR-961-5p	1.63	4.02	1.77 × 10^−4^	hsa-miR-133a ^#^

1: Mean number of sequence read counts per million reads (CPM). 2: log_2_ fold change (FC) of miRNA expression levels in mutant Huntingtin-expressing flies with respect to control flies. 3: *p* value of likelihood ratio test adjusted for multiple comparison testing using the Benjamini–Hochberg method. 4: Human orthologs are based on [18]. $: ≥70% identity with human miRNAs, #: homology at the 5′ end with human miRNAs.

## Data Availability

Small-RNA sequencing and mRNA sequencing data are available from National Center for Biotechnology Information Sequence Read Archive (NCBI SRA) under accession numbers PRJNA658796 and PRJNA982830, respectively. Other relevant data are within the manuscript, Supplementary Material, and Supporting Information files. Other datasets used and/or analyzed during the current study are available from the corresponding author on reasonable request.

## Supplementary Materials

The following supporting information can be downloaded at: https://www.mdpi.com/article/10.3390/ijms241511942/s1.
