# Supplementary material for: Dysregulated miRNA and mRNA Expression Affect Overlapping Pathways in a Huntington’s Disease Model
Source: Int J Mol Sci. 2023 Jul 26;24(15):11942. doi: 10.3390/ijms241511942 (PMC10419151; doi:10.3390/ijms241511942)
Supplement: Supplementary file 1 [file ijms-24-11942-s001.zip › ijms supplementary tables 20230711.pdf]

Supplementary material

Dysregulated miRNA and mRNA expression affect overlapping pathways in a Huntington's disease model

Nóra Zsindely[1,2], Gábor Nagy[2], Fruzsina Siági[2,3], Anita Farkas[2,3], and László Bodai[2,*]

1 Department of Genetics, Faculty of Science and Informatics, University of Szeged, H-6726 Szeged, Hungary; zsindizsn@yahoo.com

2 Department of Biochemistry and Molecular Biology, Faculty of Science and Informatics, University of Szeged, H-6726 Szeged, Közép fasor 52, Hungary

3 Doctoral School in Biology, Faculty of Science and Informatics, University of Szeged, H-6726 Szeged, Hungary

* Correspondence: bodai@bio.u-szeged.hu (L.B.)

Supplementary table S1. Characteristics of the small RNA sequencing results of the samples used in the study.

Sample	Total input reads[1]	miRNA reads[2]	Unique miRNAs[3]
Q25-1	1 904 347	410 788	176
Q25-2	2 757 414	866 899	196
Q25-3	3 858 180	1 079 675	198
Q25-4	3 271 900	1 169 939	207
Q25-5	2 475 567	968 037	199
Q25-6	3 121 385	1 185 153	211
Q25-7	3 230 513	1 123 290	209
Q120-1	3 113 643	922 047	200
Q120-2	3 143 193	1 041 266	197
Q120-3	3 309 411	1 157 384	195
Q120-4	2 066 826	1 033 405	185
Q120-5	3 376 384	1 102 404	202
Q120-6	3 680 203	1 240 629	206
Q120-7	2 878 210	997 835	200
Q120-8	2 742 327	872 116	193

1: Total number of sequence reads per sample available for bioinformatic analysis. 2: The number of sequence reads representing miRNAs per sample. 3: The number of unique miRNA species identified per sample.

Supplementary table S2. Top 10 human disease terms showing the statistically most significant enrichments in the miRNA target gene set.

Disease term	Overlap[1]	Adjusted P value[2]
Tauopathy	144/188	1.52×10^{-19}
Huntington's disease	111/136	3.59×10^{-19}
Alzheimer's disease	77/87	1.14×10^{-17}
cancer	66/74	1.17×10^{-15}
neurodegenerative disease	115/157	9.36×10^{-14}
autosomal dominant cerebellar ataxia	66/85	4.86×10^{-10}
Parkinson's disease	78/105	4.86×10^{-10}
amyotrophic lateral sclerosis	53/65	1.22×10^{-9}
viral infectious disease	40/47	1.7×10^{-8}
Epstein-Barr virus hepatitis	36/42	5.55×10^{-8}

1: Number of overlapping genes in the target gene set and the disease term set / number of genes in the disease term set. 2: P-value of Fisher’s exact test adjusted using the Benjamini-Hochberg method for correction for multiple hypotheses testing.

Supplementary Table S3. Top 10 Gene Ontology Molecular Function terms showing the statistically most significant enrichments in the miRNA target gene set.

Gene ontology term	Overlap[1]	Adjusted P value[2]
DNA binding (GO:0003677)	335/448	3.29×10^{-41}
protein kinase activity (GO:0004672)	187/228	6.03×10^{-32}
RNA polymerase II regulatory region sequence-specific DNA binding (GO:0000977)	165/200	7.12×10^{-29}
sequence-specific DNA binding (GO:0043565)	163/197	7.12×10^{-29}
transcription regulatory region sequence-specific DNA binding (GO:0000976)	165/204	4.73×10^{-27}
ubiquitin-protein transferase activity (GO:0004842)	180/231	6.71×10^{-26}
transcription factor activity, RNA polymerase II core promoter proximal region sequence-specific binding (GO:0000982)	116/134	4.71×10^{-24}
RNA binding (GO:0003723)	271/401	3.12×10^{-22}
protein serine/threonine kinase activity (GO:0004674)	135/172	5.99×10^{-20}
RNA polymerase II regulatory region DNA binding (GO:0001012)	105/127	1.26×10^{-18}

1: Number of overlapping genes in the target gene set and the GO term set / number of genes in the GO term set.
2: P-value of Fisher's exact test adjusted using the Benjamini-Hochberg method for correction for multiple hypotheses testing.

Supplementary table S4. The number of mRNA-sequencing reads per sample.

Sample	Total input reads[1]
Q25-1	5 388 896
Q25-2	7 244 627
Q25-3	6 359 422
Q25-4	6 489 262
Q120-1	6 294 197
Q120-2	6 877 695
Q120-3	6 093 111
Q120-4	6 093 786

1: Total number of sequence reads per sample available for bioinformatic analysis.
